# Comparing self-reported health interview survey and pharmacy billing data in determining the prevalence of diabetes, hypertension, and hypercholesterolemia in Belgium

**DOI:** 10.1186/s13690-023-01134-y

**Published:** 2023-06-30

**Authors:** Maria Salve Vasquez, Elly Mertens, Finaba Berete, Johan Van der Heyden, José L. Peñalvo, Stefanie Vandevijvere

**Affiliations:** 1 Department of Epidemiology and Public Health, Service of Health Information, Sciensano, Brussels, Belgium; 2 Unit of Non-Communicable Diseases, Department of Public Health, Institute of Tropical Medicine, Antwerp, Belgium

**Keywords:** Chronic diseases, Diabetes, Hypertension, Hypercholesterolemia, Pharmacy billing data, Health survey data

## Abstract

**Background:**

Administrative and health surveys are used in monitoring key health indicators in a population. This study investigated the agreement between self-reported disease status from the Belgian Health Interview Survey (BHIS) and pharmaceutical insurance claims extracted from the Belgian Compulsory Health Insurance (BCHI) in ascertaining the prevalence of diabetes, hypertension, and hypercholesterolemia.

**Methods:**

Linkage was made between the BHIS 2018 and the BCHI 2018, from which chronic condition was ascertained using the Anatomical Therapeutic Chemical (ATC) classification and defined daily dose. The data sources were compared using estimates of disease prevalence and various measures of agreement and validity. Multivariable logistic regression was performed for each chronic condition to identify the factors associated to the agreement between the two data sources.

**Results:**

The prevalence estimates computed from the BCHI and the self-reported disease definition in BHIS, respectively, are 5.8% and 5.9% diabetes cases, 24.6% and 17.6% hypertension cases, and 16.2% and 18.1% of hypercholesterolemia cases. The overall agreement and kappa coefficient between the BCHI and the self-reported disease status is highest for diabetes and is equivalent to 97.6% and 0.80, respectively. The disagreement between the two data sources in ascertaining diabetes is associated with multimorbidity and older age categories.

**Conclusion:**

This study demonstrated the capability of pharmacy billing data in ascertaining and monitoring diabetes in the Belgian population. More studies are needed to assess the applicability of pharmacy claims in ascertaining other chronic conditions and to evaluate the performance of other administrative data such as hospital records containing diagnostic codes.

**Supplementary Information:**

The online version contains supplementary material available at 10.1186/s13690-023-01134-y.

**Table:** 

**Text box 1. Contributions to the literature**
• Research has demonstrated the potential of medication indicators in monitoring diseases and risk factors in a population.
• Our work adds to the understanding of the advantages and disadvantages of using pharmacy billing data in ascertaining diabetes, hypertension, and hypercholesterolemia through its comparison with self-reported disease information.
• With the use of drug consumption information, this paper provides evidence that the Belgian compulsory health insurance database can be employed in monitoring diabetes at a population level.

## Introduction

Non-communicable diseases (NCDs), including type 2 diabetes and cardiovascular diseases, are the leading causes of mortality and morbidity globally, particularly in affluent regions like Europe where they are responsible for over 90% of all deaths [1]. According to the Global Burden of Disease Study, high serum cholesterol level, high blood pressure, and high blood glucose level are among the most prevalent metabolic risk factors explaining the high burden of NCDs, with their levels increasing globally over the last decade [2–4].

Since NCDs are of major and growing public health concern, effective disease and related risk factor surveillance is needed in monitoring their epidemiology in the population. Public health surveillance is the continuous, systematic collection and analysis, and interpretation of health-related data, including risk factors and their disease outcomes to inform prevention measures and disease management by public health authorities [5]. Active surveillance commonly involves the design and implementation of population-representative health surveys that provide periodic information on pre-defined health indicators and outcomes of the population. Health survey databases can be utilized to assess the magnitude of the problem, monitor trends in the population, provide demographic and geographic distribution of diseases, and contribute to framing the performance of policies and interventions [6, 7].

Active surveillance systems, such as nationwide health interview or examination surveys collect self-reported or measured information from a representative population sample [5]. While highly informative, the logistics of conducting a large-scale survey makes the process costly and time consuming and so national surveys are typically only organized periodically with years passing between survey cycles.

Passive health surveillance systems, on the other hand, take advantage of information collected routinely and systematically for purposes other than monitoring populations’ health [5]. Passive surveillance is a relatively inexpensive strategy able to cover large areas and populations, and to provide critical information for complementing or validating active surveillance systems. Passive health surveillance can be derived from diseases registries or hospital records, as well as health administrative databases, such as insurance health claims for medical care and treatment costs. These data collection systems have fewer logistic requirements, and are generally continuously updated, allowing for longitudinal investigation of disease outcomes and health determinants. However, they may lack certain additional individual characteristic information needed for a particular research question and may be prone to misclassification errors [8].

In Belgium, the prevalence of diseases is monitored through various surveillance efforts. For instance, the Belgian Health Interview Survey (BHIS) collects self-reported information on selected NCDs and is conducted every four or five years. Registries such as the Belgian Cancer Registry and the Belgian Diabetes Registry provide information on the incidence and prevalence of specific diseases. Sentinel surveillance systems like the Sentinel Network of General Practitioners gathers information on several diseases in Belgium.

The availability of a compulsory health insurance and the presence of a centralized databank that stores pharmacy billing records of reimbursed medicines provides an opportunity to evaluate the prevalence of selected diseases according to drug-based indicators by using information on drug prescription through the Anatomical Therapeutic Chemical (ATC) classification [9–11]. ATC assigns drugs with codes of up to five levels which describe the body system they have an effect on, therapeutic class, pharmacological actions, chemical group, and active substances. ATC then permits standardized usage of pharmaceutical products in research and drug utilization monitoring. Several studies that investigated the agreement between self-reported and pharmacy billing data relying on the ATC classification system found the latter capable of producing reliable prevalence estimates of certain NCDs, including cardiovascular diseases and diabetes [12–15]. For drugs with multiple indications, ATC classification can be used in combination with the defined daily dose (DDD) to increase the accuracy of assigning ATC codes to a particular condition [16]. This approach has been shown to be in good agreement with the self-reported diabetes, cardiovascular diseases in general, Parkinson’s disease, and thyroid disorders in Belgium for the reference year of 2008 and 2013 [17, 18].

The exploration of the potential complementarity between population-based surveys and routine administrative databases in monitoring these key cardiometabolic health indicators is important in understanding the capability of alternative data source in providing timely assessment of their health trends in the population. This entails recognizing the advantages and shortcomings of the self-reported and administrative data for these diseases and knowing the extent of association of the pharmacy billing data with different self-reported disease definitions.

This study is conducted to investigate the agreement between self-reported disease status from the Belgian Health Interview Survey (BHIS) and the pharmaceutical insurance claims extracted from the Belgian Compulsory Health Insurance (BCHI) in ascertaining the prevalence of diabetes, hypertension, and hypercholesterolemia. BCHI is further compared with the BHIS’ self-reported measures based on drug consumption since diabetes, hypertension, and hypercholesterolemia status in BCHI is determined using consumed medications. To our knowledge, these three cardiometabolic conditions which are significant risk factors of cardiovascular diseases have not been studied together in the Belgian context. In particular, this research aims to (1) quantify the agreement in the calculation of the prevalence of diabetes, hypertension, and hypercholesterolemia in Belgium using the two data sources, and (2) identify factors associated to the agreement between the survey and pharmacy billing data.

## Materials and methods

### Data sources

#### Belgian health interview surveys (BHIS)

The BHIS is a nationwide epidemiological survey, conducted by Sciensano and carried out periodically every 4 or 5 years since 1997, including a sample of around 10,000 Belgian residents per survey cycle [19]. The survey has a multi-stage cluster sampling design by which the country was stratified into the three main regions of Brussels, Flanders, and Wallonia. In a next step the regions of Flanders and Wallonia were further stratified per province, with the province of Liège split into the municipalities belonging to the German community and the other municipalities, leading to a total number of 12 geographical strata. Municipalities from each stratum were then selected, and households were subsequently chosen from these municipalities. A maximum of four household members participated in the survey. Post stratification weights were designed and applied to ensure representation of the sample in terms of age, sex, and province. Information was collected through face-to-face interview, including demographics, specific diseases and conditions, nutritional status, and through a self-administered questionnaire covering more sensitive topics such as health behaviors and lifestyle habits. Further details of the BHIS are described elsewhere [19].

#### Belgian compulsory Health insurance (BCHI)

The BCHI database includes data on reimbursed health care from the seven health insurance funds and is managed by the Intermutualist Agency (IMA-AIM) [20]. BCHI stores data of about 98% of Belgium’s national register [21]. Apart from information on reimbursed medicines from pharmacies, the BCHI database also contains a limited number of sociodemographic variables and chronic condition indicators of insured individuals in Belgium, which are based on their use of reimbursed medicines.

#### Study design

This is a cross-sectional study that investigates the agreement between BHIS 2018 and BCHI 2018 in determining the chronic disease classification and prevalence for diabetes, hypertension, and hypercholesterolemia of individuals who are at least 15 years old. HISLINK 2018 was used in the conduct of the study [22]. This is an initiative that links the BHIS 2018 to the BCHI records from 2013 and with continued update up to 2023.

### Ethics

HISLINK 2018 is a linkage of the pseudonymised BHIS and BCHI data. BHIS 2018 was organized according to the Belgian privacy legislation and approved by the Ethics Committee of the University hospital of Ghent on December, 21 2017 (advice EC UZG 2017/1454). Authorization for the data linkage of the two data sources was provided by the Belgian Information Security Committee (local reference: Deliberation No 20/204 of November 3, 2020).

### Outcome measure

Diabetes, hypertension, and hypercholesterolemia are the main conditions of interest. Three variables in BHIS were compared to BCHI-based indicators to evaluate to what extent BCHI is associated with various self-reported information. The first BHIS variable is the self-reported chronic condition (SRC) which is asked in the survey as *“During the past 12 months, have you had any of the following diseases or conditions?”*. The self-reported chronic condition and drug consumption (SRCD) is the second BHIS variable. SRCD is based on the question *“Did you take any medicines for this disease or condition during the past 12 months?”* for those who reported to have suffered from this condition. A third BHIS variable corresponds to the self-reported drug consumption obtained by asking the respondents to show the interviewers the medicines that they had used in the past 24 hours. The interviewers recorded the brand name and, if available, the national code on the package. The chronic condition was then ascertained from the ATC of consumed medications (CATC). Categorization of disease status for CATC relies solely on whether a drug with ATC associated with a chronic condition (Table 1) was taken in the given timeframe.

**Table 1:** ATC and description of drugs associated to diabetes, hypertension, and hypercholesterolemia

Condition	ATC	Medication
Diabetes	A10A A10B	Insulins and analogues Blood glucose lowering drugs, excluding insulins
Hypertension	C02 C03 C07 C08 C09	Antihypertensive Diuretics Beta-blocking agents Calcium channel blockers Agents acting on the renin-angiotensin system
Hypercholesterolemia	C10	Lipid modifying agents

Pseudodiagnosis for a group of diseases, including diabetes, was developed by an experts group for the National Institute for Health and Disability Insurance (NIHDI) using algorithm based on the use of reimbursed medicines [16]. These pseudodiagnoses were contained in the HISLINK database used for this study. Since hypertension and hypercholesterolemia were not yet included, the same case-finding algorithm was applied by the researchers. That is, a one-year cumulative DDD of at least 90 for all the consumed disease-specific medications (see Table 1) assigns an individual to a chronic condition. For each person, the total DDD for a particular chronic condition is computed as

$$Total DDD=\sum _{i=1}^{n}{Quantity}_{i}\times {DPP}_{i}$$

where *Quantity*_*i*_ = quantity of the *i*^*th*^ reimbursed drug;

*DPP*_*i*_ = daily defined doses per package of the *i*^*th*^ reimbursed drug; and.

*n* = number of drug reimbursements made in a year.

### Statistical analysis

The weighted prevalence of diabetes, hypertension, and hypercholesterolemia in Belgium in 2018 was calculated according to the different definitions in BHIS and BCHI. The percentage of individuals with disease status classification in agreement between the data sources was estimated. The Cohen’s kappa coefficient (*κ*) was computed to investigate the degree of agreement between the two data sources’ classification. A kappa coefficient of 0 to 0.20 is interpreted as no agreement; 0.21 to 0.39 as minimal; 0.40 to 0.59 as weak; 0.60 to 0.79 as moderate; 0.80 to 0.90 as strong; and above 0.90 as almost perfect agreement [23]. Accompanying the kappa coefficient is a corresponding 95% uncertainty obtained using the *epi.kappa* function of the *epiR* package in the R software.

Each disease variable in the BHIS was compared to its corresponding disease indicator in the BCHI. For each comparison and with BHIS as the reference standard, sensitivity, specificity, negative predictive value (NPV), and positive predictive value (PPV) were calculated to understand the degree to which BCHI accurately measures disease occurrence. These measures and their 95% confidence interval were generated using the *epi.tests* function of the *epiR* package in R.

Multivariable logistic regression that takes survey weights and design into account was performed for each chronic condition to identify the factors associated to the agreement between the two data sources. The fit of the models was assessed using the Hosmer-Lemeshow goodness-of-fit test. The dependent variable is defined as the agreement in disease status classification. It is a binary variable that indicates whether there is consistency in the reported disease status in BHIS and the identified disease status in BCHI. The two categories of the dependent variable are: similar and dissimilar classification. Specifically, if an individual is classified in both data sources as having a particular disease or if there is an agreement in both data sources that a given disease is not present, then there is similar classification. If otherwise, the value of the dependent variable corresponds to dissimilar classification.

Independent variables from the BHIS include age categories (15–34, 35–54, 55–74, 75+), gender (male versus female), educational attainment (no diploma or primary education, lower secondary, higher secondary, higher education), multimorbidity (suffering from at least two chronic diseases versus less than 2), nationality (Belgian, non-Belgian EU, non-Belgian non-EU), household income quintile (Q1, Q2, Q3, Q4, Q5), region (Brussels, Flanders, Wallonia), and subjective health (good to very good versus very bad to fair). Age was categorized since it is of interest to assess the effect of the different classification, from younger to older levels. These variables were chosen as they were deemed to be potential risk factors of disagreement between BHIS and BCHI.

The data management and statistical analyses were performed, respectively, in SAS 9.4 and R 4.1.2.

## Results

### Overview of the study sample

The sample in BHIS consists of survey participants aged 15 years and older (*n* = 9753). Of these, 9167 individuals (94% of the BHIS participants) were successfully linked to BCHI 2018.

A summary of the study population’s characteristics is provided in Table 2. The study population consists of a slightly higher proportion of women and residents in the 35–54 years old category, with about 11% of the population aged 75 and older. The majority of the population belongs to a household with at least a higher secondary as the highest level of education and to a higher income group of fourth and fifth income quintile. The study population is also characterized by predominantly Belgian nationals (90%) residing in the Flemish region (57%). In addition, 23% participants reported fair to very bad subjective health, and 15% suffered from at least two chronic diseases.

The frequency and weighted prevalence rates of diabetes, hypertension, and hypercholesterolemia for the different disease definitions are summarized in Table 3. Among the BHIS variables, SRC produced the highest prevalence estimate for diabetes (5.9%) and hypercholesterolemia (18.1%) while CATC produced the highest prevalence estimate for hypertension (22.2%). Considering all disease definitions, prevalence estimates for hypercholesterolemia showed a dispersion (*range* = 5.4, *σ*^*2*^ = 4.6) ranging from 12.7% to 18.1%.

Relative to the other chronic conditions, estimates for hypertension had the highest variability (*range* = 9.2, *σ*^*2*^ = 13.2) and ranged from 15.4% to 24.6%. On the contrary, the weighted prevalence of diabetes in the population had the lowest variability (*range* = 0.5, *σ*^*2*^ = 0.1) compared to hypertension and hypercholesterolemia.

**Table 2:** Characteristics of the Belgian population (*n* = 9,167) included in the study

Characteristic	Frequency ^a^
***Age (years)***	
15–34	2,248 (29)
35–54	3,030 (32)
55–74	2,833 (28)
75+	1,056 (11)
***Gender***	
Men	4,370 (48)
Women	4,797 (52)
***Highest educational level within the household***	
No diploma or primary education	693 (6)
Lower secondary	1,237 (13)
Higher secondary	2,782 (33)
Higher	4,304 (48)
***Household income***	
Quintile 1	1,089 (12)
Quintile 2	1,253 (15)
Quintile 3	1,555 (20)
Quintile 4	1,924 (25)
Quintile 5	1,922 (28)
***Nationality***	
Belgian	7,938 (90)
Other EU nationalities	702 (5)
Non-EU nationalities	523 (5)
***Region***	
Flanders	3,581 (57)
Brussels	2,321 (10)
Wallonia	3,265 (33)
***Subjective health perception***	
Good - Very Good	5,708 (77)
Fair - Very Bad	1,831 (23)
***Multimorbidity***	
With multimorbidity	1,483 (15)
Without multimorbidity	7665 (85)

^a^ The figures correspond to the number of respondents belonging to a category and their respective survey-weighted percentages

**Table 3:** Survey-weighted prevalence of chronic conditions among the Belgian population (2018) based on BHIS and BCHI definitions ^a^

Variables	Diabetes		Hypertension		Hypercholesterolemia	
	*n* (%)	*Diff* ^b^	*n* (%)	*Diff* ^b^	*n* (%)	*Diff* ^b^
BHIS						
SRC	609 (5.9)	0.1	1697 (17.6)	-7.0	1704 (18.1)	1.9
SRCD	556 (5.4)	-0.4	1490 (15.4)	-9.2	1210 (12.7)	-3.5
CATC	542 (5.4)	-0.4	2167 (22.2)	-2.4	1319 (13.6)	-2.6
BCHI	591 (5.8)		2434 (24.6)		1572 (16.2)	

^a^ The figures correspond to the number of individuals with chronic condition and their respective weighted prevalence

^b^ Percent difference between the BHIS and BCHI disease prevalence

Abbreviations: BCHI, Belgian compulsory health insurance; BHIS, Belgian Health Interview Survey; CATC, chronic condition ascertained from the ATC of consumed medications; SRC, self-reported chronic condition; SRCD, self-reported chronic condition and drug consumption

Comparing the BCHI to the BHIS prevalence estimates, the former underestimated the latter’s SRC for diabetes and hypercholesterolemia (Table 3). The BCHI for hypertension overestimated the prevalence estimates of all three BHIS figures with the smallest difference obtained between BCHI and CATC.

Table 4 presents the percent agreement and kappa coefficient between the prevalence of cardiometabolic conditions ascertained by BHIS or BCHI databases. The agreement between the two data sources is consistently high for diabetes. Specifically, about 98% of the individuals were similarly classified in the pairwise comparison of SRC, SRCD, and CATC with BCHI, and with the kappa coefficient suggesting a substantial agreement between the data sources. Conversely, the percent agreement and the kappa coefficient between BCHI and hypertension’s SRC (*% agree* = 86.0%; $\widehat{\kappa }$=0.60) and SRCD (*% agree* = 86.6%; $\widehat{\kappa }$=0.61) were lower than that of CATC (*% agree* = 93.4%; $\widehat{\kappa }$=0.82). For both hypertension and hypercholesterolemia, only CATC reached a strong agreement with BCHI as SRC and SRCD has either a weak or moderate agreement. Hypercholesterolemia’s kappa coefficient is particularly at its lowest when BCHI is compared with SRC.

**Table 4:** Measures of validity between the BCHI and each of the three BHIS variables ^a^

Variables	Agreement (%)	Kappa (95% CI)	Sensitivity (%) (95% CI)	Specificity (%) (95% CI)	PPV (%) (95% CI)	NPV (%) (95% CI)
Diabetes						
SRC	97.6	0.80 (0.78, 0.83)	80.5 (77.1, 83.5)	98.8 (98.6, 99.0)	82.9 (79.6, 85.9)	98.6 (98.3, 98.8)
SRCD	97.9	0.82 (0.80, 0.85)	86.0 (82.8, 88.8)	98.7 (98.4, 98.9)	80.9 (77.5, 84.0)	99.1 (98.9, 99.3)
CATC	97.9	0.82 (0.80, 0.85)	86.9 (83.8, 89.6)	98.6 (98.3, 98.8)	79.7 (76.2, 82.9)	99.2 (99.0, 99.4)
Hypertension						
SRC	86.0	0.60 (0.58, 0.62)	83.9 (82.1, 85.6)	86.5 (85.7, 87.2)	58.5 (56.5, 60.5)	95.9 (95.4, 96.4)
SRCD	86.6	0.61 (0.59, 0.63)	90.5 (88.9, 92.0)	85.9 (85.0, 86.6)	55.4 (53.4, 57.4)	97.9 (97.5, 98.2)
CATC	93.4	0.82 (0.81, 0.84)	92.1 (90.9, 93.2)	93.7 (93.1, 94.3)	82.0 (80.4, 83.5)	97.5 (97.1, 97.8)
Hypercholesterolemia						
SRC	85.4	0.51 (0.48, 0.53)	57.1 (54.7, 59.5)	92.0 (91.3, 92.6)	61.9 (59.5, 64.3)	90.3 (89.6, 91.0)
SRCD	89.9	0.61 (0.59, 0.63)	76.8 (74.3, 79.1)	91.9 (91.3, 92.5)	59.1 (56.6, 61.5)	96.3 (95.9, 96.7)
CATC	95.3	0.82 (0.81, 0.84)	93.2 (91.7, 94.5)	95.6 (95.2, 96.1)	78.2 (76.1, 80.2)	98.8 (98.5, 99.0)

^a^ BHIS is used as the reference in the computation of the sensitivity, specificity, PPV, and NPV

Abbreviations: BCHI, Belgian compulsory health insurance; BHIS, Belgian Health Interview Survey; CATC, chronic condition ascertained from the ATC of consumed medications; SRC, self-reported chronic condition; SRCD, self-reported chronic condition and drug consumption; PPV, positive predictive value; NPV, negative predictive value

The resulting measures of concordance for the pairing of BCHI with each of SRC, SRCD, and CATC are presented in Table 4. Compared to the other chronic conditions, diabetes recorded the highest proportion of agreement of more than 97% between the BCHI and each of the three BHIS variables. The chance that a person does not have diabetes in BCHI given a diabetes-free assessment in BHIS is at least 98%. The sensitivity of BCHI in identifying diabetes cases ranged from 80.5 to 86.9% while its PPV from 79.7 to 82.9%. Hypertension has at least 95% of NPV, and its PPV for SRC and SRCD is 58.5% and 55.4%, respectively. When compared with the CATC of hypertension, BCHI yielded high measures of sensitivity, specificity, PPV, and NPV which are equivalent to 92.1%, 93.7%, 82.0%, and 97.5%, respectively. Similarly, computed measures (sensitivity = 93.2%; specificity = 95.6%; PPV = 78.2%; NPV = 98.8%) for hypercholesterolemia are consistently high when BCHI is compared to CATC. For hypercholesterolemia, BCHI has the low sensitivity of 57.1% to SRC and a low PPV of 59.1% to SRCD.

The result of the multivariable survey-weighted logistic regression is presented in the additional file. The Hosmer-Lemeshow test indicates that the models fit well (p-values > 0.05). Several factors are significantly associated to the agreement between BHIS and BCHI with the magnitude of associations varying across diseases and case definitions.

For diabetes, the set of factors that are significantly associated with the agreement between the BHIS and the BCHI vary across the former’s disease definition. Older age categories are consistently associated with lower odds of agreement between BCHI and the BHIS’ disease definition of SRC, SRCD, and CATC. Furthermore, individuals with at least two chronic conditions are less likely to have agreement in their disease status relative to those without multimorbidity.

For hypertension, agreement between the SRC and the BCHI is associated with gender, age, and health perception. The odds of agreement between SRC and BCHI is higher among women (OR 1.29; 95% CI: 1.05, 1.58) than men and among those with good to very good subjective health (OR 1.65; 95% CI: 1.32, 2.08). Compared to those who are 35 to 54 years old, people belonging to the older age categories have lower odds of agreement between the two data sources.

For hypercholesterolemia, agreement between SRC and BCHI is significantly positively associated with female gender (OR 1.32; 95% CI: 1.09, 1.59), younger age (OR 3.71; 95% CI: 2.46, 5.60), and positive subjective health perception (OR 1.47; 95% CI: 1.18, 1.84). A positive association is likewise observed for the female gender (OR 1.38; 95% CI: 1.11, 1.72), younger age (OR 12.91; 95% CI: 4.87, 34.22), and positive health perception (OR 1.39; 95% CI: 1.08, 1.78) between SRCD and BCHI. The young age group of 15 to 34 years (OR 6.06; 95% CI: 1.76, 20.84) and the positive subjective health perception (OR 1.51; 95% CI: 1.07, 2.13) have a similar association to the agreement between CATC and BCHI.

## Discussion

This study investigated the agreement between the population-based interview survey’s self-reported chronic conditions, assessed in different ways, and administrative health insurance records for drug-treated diabetes, hypertension, and hypercholesterolemia. BCHI disease indicators were generated using information on drug consumption. The nature of these indicators prompted its comparison to not just the SRD but also to the self-reported drug-based SRCD and CATC. Briefly, the results showed strong agreement between the BHIS and BCHI for diabetes, and poor agreement and inconsistent direction of disease prevalence for hypertension and hypercholesterolemia. In addition, agreement between BHIS and BCHI for hypercholesterolemia were more frequently observed among women, positive health perception and younger ages.

The administrative health insurance claims for medications can be an alternative data source in the estimation of diabetes prevalence in the population as the analysis showed high level of agreement and small deviation in the computed prevalence estimates between the two data sources. Using the 2018 Belgian Health Examination Survey (BHES), a study that objectively measured the blood pressure, blood cholesterol levels, and fasting plasma glucose and HbA1C in a subset and thus small sample of BHIS participants, a small difference in the estimates of self-reported condition and medication use was also found, along with the observation of a large proportion (85.6%) of the people who are at least 18 years old and suffering from diabetes were actually taking medication [24]. This finding is also consistent with the result of other studies [12, 17, 25–27]. The small difference in the estimated prevalence between BHIS and BCHI could possibly be attributed to the impact that this condition has to an individual’s quality of life and healthcare expenses and the burden that it likewise renders to the healthcare system. Meanwhile, the slight discrepancy in the estimates could be due to the sensitivity of the case-finding algorithm and the DDD threshold used in identifying people with diabetes [17]. This may also be explained by the shortcomings of both data sources in the same direction. For example, BHIS may underestimate because of false negative reporting while BCHI missed diabetes patients who do not take medication or who took medication but with amount not reaching the threshold of the case-finding algorithm.

Insurance claims produced an overestimated prevalence of self-reported hypertension in the population. The overestimation could be due to the common use of blood pressure lowering medications in the secondary prevention of cardiovascular diseases [28], as well as the use of antihypertensives for multiple indications in cardiovascular and chronic diseases [15]. In fact, BHES reported that 45% of the individuals with high or potentially high blood pressure actually reported that they have this condition [24]. The indistinguishability of conditions to which the medications were indicated and the nature of the case-finding algorithm resulted to the poor agreement between the insurance claims and the self-reported hypertension in our study. However, better concordance between administrative and self-reported data for hypertension were observed in cases where the former is defined using medical or diagnostic codes. Muggah et al. used hospital and physician billing codes and estimated a κ coefficient of 0.66 between the administrative and self-reported data for this condition [29]. Using the International Classification of Diseases, Ninth Revision, Clinical Modification (ICD-9-CM) code 401 for hypertension, Lix et al. tried various algorithms consisting of physician data, hospital data, prescription drug data, and their combinations and compared it with survey records [26]. Their study found that using one year of data, the estimated κ coefficients ranged from 0.54 to 0.70, with the maximum value computed from an algorithm with a combination of at least one hospital separations or at least one physician billing or at least two prescription drug records.

The pharmacy billing data has a weak agreement with the self-reported data on the prevalence of hypercholesterolemia. This disagreement could be partially explained by the large underdiagnoses of hypercholesterolemia as in Belgium, only one in three individuals aged 18 and older with high or potentially high serum cholesterol was aware and reported having this condition according to the analyses of the BHES [24]. Another factor could be the poor adherence to lipid-lowering drugs, particularly statins [30–32] which can be used in treating other conditions [33] or may not be appropriate for everybody with hypercholesterolemia [34]. In addition, participants with multimorbidity in polypharmacy regimes may misreport their diagnosis often because of the pleiotropic effect of statins. The disagreement between self-reported and claims-based prevalence of hypercholesterolemia is challenging to explain but may reflect the reversibility of elevated blood cholesterol or an initial treatment with lifestyle medication. Nevertheless, the analysis showed that better agreement is found if drug-based self-reported data are considered and especially if information is based on the registration of the brand names of the consumed drugs.

Sociodemographic and health factors affect the agreement between the self-reports and insurance claims. The strength of association of these factors differ across chronic conditions but in most of our comparisons, high agreement is observed among younger individuals, among those with positive subjective health perception, and among people without comorbid conditions. These results are consistent with the findings of other studies [17, 35–37]. In particular, Wu et al. discovered in their analysis the association of old age to the disagreement between self-reports and claims data as well as the data sources’ concordance among highly educated participants [37]. Chiu et al. also confirmed in their investigation of the factors associated to the difference in self-reports and ICD-9 codes from medical records that under-reporting of a disease is more likely among respondents who are older or have poor health [36]. The association of old age and multimorbidity to the disagreement in different data sources has also likewise been concluded in the studies conducted by Berete et al. and Lix et al. [17, 35]. The disagreement in the self-reported and insurance-based disease status among the elderlies could be explained by multimorbidity, the development and diagnosis of various chronic ailments, and the resulting consumption of an assortment of medications. Additionally, lapses in memory and utilization of drugs that can be used in treating multiple conditions could also contribute to the discrepancies between the two data sets.

The PPV and the NPV are among the estimates computed to assess the performance of the pharmacy billing data. However, the comparison of the PPV and the NPV between the three diseases is tricky because these estimates are influenced by the magnitude of the prevalence of the disease.

It is important to understand the temporal invariance in the relationship between administrative and survey data in recommending the former as a potential alternative or supplement to the latter. This ensures consistency in measurements and precision in conclusion. Although longitudinal analysis was not performed in this study, our result on the strong agreement between the two data sets in estimating the prevalence of diabetes is consistent with that observed in the linked BHIS and BCHI 2013 [17]. The conformity in the conclusion further strengthens the utility of the claims database as a means of diabetes surveillance in the population.

This study has several limitations. Diagnostic codes that could have been used in combination with or a point of comparison to pharmacy claims is absent in the database and therefore not included in the analysis. In place of an accurate diagnosis, the self-reported chronic condition from the survey was treated as the reference standard. However, this data is not free from bias and could also produce under- or over-estimated disease prevalence [38]. Potential sources of bias in the self-reported health data include measurement bias, recall bias, and social desirability bias.

Despite the limitations, this research has its strength in the size and representativeness of the study population. The capacity of the healthcare system to link individuals from these two databases using the unique national register number avoids linkage errors. This study also computed various agreement measures and identified factors associated with the agreement of the two data sources. Furthermore, the comparison of pharmacy claims to three different self-reported disease definitions provided better understanding of the estimates generated from the insurance database.

The small variation in the prevalence estimates, the high percentage of agreement and the strong concordance between the self-reported and the pharmacy billing data suggests the potential of the BCHI as a resource for the surveillance of diabetes in Belgium. On the contrary, the BCHI’s biased prevalence estimates and relatively low measures of agreement for the self-reported hypertension and hypercholesterolemia provides evidence of the challenges of using the pharmacy billing data in monitoring these conditions. The nature of these diseases and the applicability of their medications to a wide array of conditions calls for the adoption of an algorithm that involves medical codes in the ascertainment of health conditions.

## Conclusion

This study demonstrated the value of pharmacy billing data in ascertaining and monitoring diabetes in the Belgian population. The agreement between the self-reported and the administrative insurance data is higher for chronic conditions that have specific treatment strategies and lower for those with non-exclusive set of medications such that in the case of hypertension. The pharmacy billing data similarly has poor agreement with the self-reported hypercholesterolemia. The association of older adults to the disagreement between the data sources must be taken in consideration in the conduct of epidemiological studies on this population. Medical codes with information on diagnoses and health procedures is an advantageous addition to the linkage of the pharmacy billing and self-reported health data. Its inclusion would provide specific information on health conditions and complement currently available healthcare information. Thus,to further broaden the investigation of the performance of administrative data in ascertaining chronic conditions, this study suggests for an effort to link pharmacy billing data with diagnostic information from primary care data.

## Electronic supplementary material

Below is the link to the electronic supplementary material.

Additional file: Results of the regression analysis

## Data Availability

The HISLINK data analyzed in this study is not publicly available due to the General Data Protection Regulation (GDPR) in the processing of personal data. The data can be accessed only upon request to the Social Security and Health Chamber of the Information Security Committee (https://www.ehealth.fgov.be/ehealthplatform/nl/informatieveiligheidscomite).

## Abbreviations

ATC: Anatomical Therapeutic Chemical
BCHI: Belgian Compulsory Health Insurance
BHES: Belgian Health Examination Survey
BHIS: Belgian Health Interview Survey
CATC: Chronic condition ascertained from the ATC of the reported drugs
DDD: Defined Daily Dose
SRC: Self-reported chronic condition
SRCD: Self-reported chronic condition and drug consumption

## References

[1] Global burden of 369 diseases and injuries in 204 countries and territories, 1990–2019: a systematic analysis for the Global Burden of Disease Study 2019. Lancet. 2020;396(10258):1204–1222. doi:10.1016/S0140-6736(20)30925-9. 10.1016/S0140-6736(20)30925-9 PMC7567026 33069326

[2] Tran KB, Lang JJ, Compton K (2022). The global burden of cancer attributable to risk factors, 2010–19: a systematic analysis for the global burden of Disease Study 2019. The Lancet.

[3] Zheng J, Wang J, Zhang Y, et al. The Global Burden of Diseases attributed to high low-density lipoprotein cholesterol from 1990 to 2019. Front Public Health. 2022;10. 10.3389/fpubh.2022.891929. https://www.frontiersin.org/articles/. 10.3389/fpubh.2022.891929 PMC9424500 36051998

[4] Murray CJL, Aravkin AY, Zheng P (2020). Global burden of 87 risk factors in 204 countries and territories, 1990–2019: a systematic analysis for the global burden of Disease Study 2019. The Lancet.

[5] Nsubuga P, White M, Thacker S et al. Public health surveillance: a tool for targeting and monitoring interventions. In: Disease Control Priorities in developing countries. 2nd ed. Oxford University Press. https://www.ncbi.nlm.nih.gov/books/NBK11770/.

[6] Lix LM, Ayles J, Bartholomew S (2018). The canadian chronic Disease Surveillance System: a model for collaborative surveillance. Int J Popul Data Sci.

[7] Martin GS (2008). The essential nature of healthcare databases in critical care medicine. Crit Care.

[8] Funk MJ, Landi SN (2014). Misclassification in administrative claims data: quantifying the impact on treatment effect estimates. Curr Epidemiol Rep.

[9] World Health Organization. Introduction to Drug Utilization Research. ; 2003. Accessed July 27, 2022. https://apps.who.int/iris/handle/10665/42627.

[10] Hollingworth S, Kairuz T. Measuring Medicine Use: applying ATC/DDD Methodology to Real-World Data. Pharm (Basel). 2021;9(1). 10.3390/pharmacy9010060. 10.3390/pharmacy9010060 PMC8006033 33802774

[11] WHO Collaborating Centre for Drug Statistics Methodology. ATC/DDD Index. WHO Collaborating Centre for Drug Statistics Methodology. Accessed April 1., 2022. https://www.whocc.no/atc_ddd_index/.

[12] Chini F, Pezzotti P, Orzella L, Borgia P, Guasticchi G (2011). Can we use the pharmacy data to estimate the prevalence of chronic conditions? A comparison of multiple data sources. BMC Public Health.

[13] Haapea M, Miettunen J, Lindeman S, Joukamaa M, Koponen H (2010). Agreement between self-reported and pharmacy data on medication use in the Northern Finland 1966 Birth Cohort. Int J Methods Psychiatr Res.

[14] Matsumoto M, Harada S, Iida M (2021). Validity Assessment of Self-reported Medication Use for Hypertension, Diabetes, and Dyslipidemia in a pharmacoepidemiologic study by comparison with Health Insurance Claims. J Epidemiol.

[15] Huber CA, Szucs TD, Rapold R, Reich O (2013). Identifying patients with chronic conditions using pharmacy data in Switzerland: an updated mapping approach to the classification of medications. BMC Public Health.

[16] EPS R13 - FLAGS Release 20190201 NL, Accessed. February 10, 2022. https://aim-ima.be/IMG/pdf/eps_r13_-_flags_release_20190201_nl_-_vs2.pdf.

[17] Berete F, Demarest S, Charafeddine R, Bruyère O, Van der Heyden J (2020). Comparing health insurance data and health interview survey data for ascertaining chronic disease prevalence in Belgium. Archives of Public Health.

[18] Vaes B, Ruelens C, Saikali S (2018). Estimating the prevalence of diabetes mellitus and thyroid disorders using medication data in Flanders, Belgium. Eur J Pub Health.

[19] Demarest S, Van der Heyden J, Charafeddine R, Drieskens S, Gisle L, Tafforeau J (2013). Methodological basics and evolution of the belgian health interview survey 1997–2008. Archives of Public Health.

[20] Farmanet Metadata. IMA-AIM, Accessed. September 6, 2022. https://metadata.ima-aim.be/nl/app/bdds/Fu.

[21] Population Metadata. IMA-AIM. http://metadata.ima-aim.be/nl/app/bdds/Pp.

[22] HISLINK. 2018. Accessed September 5, 2022. https://www.ehealth.fgov.be/ehealthplatform/file/view/AXW7dlaDl9vUUfvGGe52?filename=20-204-n382-HISLINK%202018.pdf.

[23] McHugh ML (2012). Interrater reliability: the kappa statistic. Biochem Med (Zagreb).

[24] Van der Heyden J, Nguyen D, Renard F et al. Belgian Health Examination Survey 2018. Accessed September 16, 2022. https://www.sciensano.be/sites/default/files/report_hes_masterfile_nl_final_1.pdf.

[25] Rector TS, Wickstrom SL, Shah M (2004). Specificity and sensitivity of claims-based algorithms for identifying members of Medicare + Choice health plans that have chronic medical conditions. Health Serv Res.

[26] Lix L, Yogendran M, Mann J. Defining and Validating Chronic Diseases: An Administrative Data Approach – An Update with ICD-10-CA. Published online January 1, 2006.

[27] Fortin M, Haggerty J, Sanche S, Almirall J (2017). Self-reported versus health administrative data: implications for assessing chronic illness burden in populations. A cross-sectional study. CMAJ Open.

[28] Rahimi K, Bidel Z, Nazarzadeh M (2021). Pharmacological blood pressure lowering for primary and secondary prevention of cardiovascular disease across different levels of blood pressure: an individual participant-level data meta-analysis. The Lancet.

[29] Muggah E, Graves E, Bennett C, Manuel DG (2013). Ascertainment of chronic diseases using population health data: a comparison of health administrative data and patient self-report. BMC Public Health.

[30] Zodda D, Giammona R, Schifilliti S. Treatment strategy for Dyslipidemia in Cardiovascular Disease Prevention: Focus on Old and New Drugs. Pharm (Basel). 2018;6(1). 10.3390/pharmacy6010010. 10.3390/pharmacy6010010 PMC5874549 29361723

[31] Gatwood J, Bailey JE. Improving Medication Adherence in Hypercholesterolemia: Challenges and Solutions. Vol 10.; 2014. doi:10.2147/VHRM.S56056. 10.2147/VHRM.S56056 PMC4226449 25395859

[32] Man REK, Gan AHW, Fenwick EK (2019). Prevalence, determinants and association of unawareness of diabetes, hypertension and hypercholesterolemia with poor disease control in a multi-ethnic asian population without cardiovascular disease. Popul Health Metrics.

[33] Liao JK, Laufs U (2005). Pleiotropic effects of statins. Annu Rev Pharmacol Toxicol.

[34] Krähenbühl S, Pavik-Mezzour I, von Eckardstein A (2016). Unmet needs in LDL-C lowering: when Statins won’t do!. Drugs.

[35] Lix LM, Yogendran MS, Shaw SY, Burchill C, Metge C, Bond R (2008). Population-based data sources for chronic disease surveillance. Chronic Dis Can.

[36] Chiu CJ, Huang HM, Lu TH, Wang YW (2018). National health data linkage and the agreement between self-reports and medical records for middle-aged and older adults in Taiwan. BMC Health Serv Res.

[37] Wu CS, Lai MS, Gau SSF, Wang SC, Tsai HJ (2014). Concordance between patient self-reports and Claims Data on Clinical Diagnoses, Medication Use, and Health System utilization in Taiwan. PLoS ONE.

[38] Pelgrims I, Devleesschauwer B, Doggen K, et al. Validity of self-reported data to assess the prevalence of overweight, hypertension and cholesterol. Eur J Pub Health. 2021;31(Supplement3). ckab164.750. 10.1093/eurpub/ckab164.750.

